# Foramen Ovale Measurements and Venous Hemodynamic Changes Assessed by Inferior Vena Cava Doppler Parameters in Early- and Late-Onset Fetal Growth Restriction

**DOI:** 10.3390/jcm15030980

**Published:** 2026-01-26

**Authors:** Merve Ayas Ozkan, Halis Doğukan Ozkan, Ruken Dayanan, Hilal Sarı, Furkan Akın, Gülşah Dağdeviren, Ali Turhan Çağlar

**Affiliations:** 1 Depatment of Obstetrics and Gynecology, Division of Perinatology, Ankara Etlik City Hospital, 06170 Ankara, Turkey; rukendayanan@gmail.com (R.D.); hilalsarii@icloud.com (H.S.); furkanakin@gmail.com (F.A.); dagdevirengulsah@hotmail.com (G.D.); turhan_caglar@yahoo.com (A.T.Ç.); 2 Dogukan Ozkan Clinic, Private Practice, Obstetrics and Gynecology, 06510 Ankara, Turkey; hdogukanozkan@gmail.com

**Keywords:** fetal growth restriction, foramen ovale, vena cava inferior, Doppler ultrasonography, perinatal outcome

## Abstract

**Background**: Fetal growth restriction (FGR) is a major contributor to adverse perinatal outcomes and is primarily driven by placental insufficiency and chronic fetal hypoxia. While arterial Doppler abnormalities are widely used in clinical surveillance, less is known about venous hemodynamics and intracardiac structural adaptations in FGR. In particular, the clinical relevance of foramen ovale (FO) morphometry and inferior vena cava (IVC) Doppler parameters in different FGR phenotypes remains incompletely understood. This study aimed to evaluate FO measurements and IVC Doppler indices in early- and late-onset FGR and to investigate their associations with adverse perinatal outcomes. **Methods**: This prospective observational study included 240 singleton pregnancies: 120 fetuses with FGR and 120 gestational age-matched appropriate-for-gestational-age controls. FGR was defined according to Delphi consensus criteria and classified as early onset (<32 weeks) or late onset (≥32 weeks). Ultrasonographic assessment included FO and right atrium dimensions, FO-to-right atrium (FO/RA) ratio, IVC diameter, and IVC Doppler indices (pulsatility index [PI], preload index [PLI], and peak velocity index for veins [PVIV]). A composite adverse perinatal outcome (CAPO) was recorded. Receiver operating characteristic (ROC) curve analysis and multivariable logistic regression were performed. **Results**: Compared with controls, fetuses with FGR exhibited significantly smaller FO dimensions, lower FO/RA ratios, reduced IVC diameters, and higher IVC Doppler indices (all *p* < 0.05). The FO/RA ratio demonstrated the highest discriminative performance for CAPO (AUC 0.722). In multivariable analysis, a 0.1-unit increase in the FO/RA ratio was independently associated with a reduced risk of CAPO (OR 0.57), whereas higher IVC PI values were associated with an increased risk (OR 2.64). IVC Doppler alterations were less pronounced in early-onset FGR. **Conclusions**: FO morphometry and IVC Doppler parameters reflect complementary stages of fetal cardiovascular adaptation in fetal growth restriction, with FO changes representing early adaptive responses and IVC Doppler alterations indicating more advanced hemodynamic compromise, and this may provide additional value for perinatal risk stratification.

## 1. Introduction

Fetal growth restriction (FGR) is one of the main causes of perinatal morbidity and mortality [1]. Population-based data specifically reporting the prevalence of FGR in Türkiye are currently limited; however, available national demographic and health statistics provide important contextual information on fetal growth patterns in the population. Although small-for-gestational-age (SGA) status and low birth weight are not synonymous with FGR, reported rates of these indicators may offer indirect insight into the regional perinatal growth profile [2]. When interpreted alongside global data indicating that FGR affects approximately 8–10% of pregnancies worldwide, such information helps contextualize the burden of impaired fetal growth within the regional setting [3,4]. It is known that the main cause of FGR is placental insufficiency and impaired fetoplacental circulation [5]. In FGR, not only arterial Doppler abnormalities but also adaptations at the fetal heart and central venous system levels are components of the pathophysiology [6,7]. These adaptive responses occur so that the fetus can maintain adequate oxygenation to its vital organs under chronic hypoxemia and increased placental resistance [8]. In this context, the evaluation of venous and intracardiac parameters has gained interest as a complementary approach to traditional arterial Doppler monitoring.

The fetal venous system provides direct information about cardiac preload, central venous pressure, and myocardial compliance [9]. In particular, inferior vena cava (IVC) Doppler waveforms reflect the interaction between venous return and right atrial function and are sensitive to changes in fetal cardiac loading conditions [10]. Changes in venous Doppler indices have been associated with advancing fetal distress and adverse perinatal outcomes [11,12]. However, unlike ductus venosus Doppler, which is routinely included in clinical management algorithms, IVC Doppler parameters have been less studied in pregnancies diagnosed with FGR.

Parallel to venous functional changes, structural and morphometric adaptations of the fetal heart may also occur in growth-restricted fetuses. The foramen ovale (FO) is an important intracardiac shunt that facilitates the preferential flow of oxygenated blood to the left atrium and cerebral circulation. Therefore, changes in FO size and morphology may reflect alterations in intracardiac flow distribution and adaptive cardiac remodeling [13,14]. Studies have shown that FO dimensions are reduced in fetuses with FGR and that a decrease in FO size is associated with adverse perinatal outcomes, suggesting that FO morphometry may serve as an additional marker of fetal hemodynamic dysfunction [13,15]. FO measurements are not routinely assessed, and their relationship with venous Doppler changes has not been extensively investigated.

Early-onset and late-onset FGR have distinct clinical and pathophysiological causes [16]. Early-onset FGR is typically characterized by severe placental disease, progressive arterial and venous Doppler abnormalities, and risk of fetal decompensation. Late-onset FGR, on the other hand, usually presents with milder hemodynamic changes and arterial Doppler findings [17]. Whether venous Doppler indices of the IVC and FO morphometric parameters differ between these two FGR phenotypes has not been sufficiently investigated. FO and IVC are complementary structures reflecting early intracardiac adaptation and late hemodynamic deterioration in fetal circulation. The combined evaluation of these two structures may allow for a more comprehensive examination of the different stages of fetal cardiovascular adaptation in FGR. Therefore, the aim of this study is to evaluate FO measurements and venous hemodynamic changes assessed by IVC Doppler parameters in fetuses with early- and late-onset FGR and to investigate their relationship with perinatal outcomes.

## 2. Materials and Methods

This study is a prospective, observational, and comparative study conducted between September 2024 and September 2025, following ethical approval. The study has been reported in accordance with the STROBE (Strengthening the Reporting of Observational Studies in Epidemiology) statement. The study protocol was approved by the relevant local ethics committee (AEŞH-BADEK-2024-256, date: 3 April 2024), and written informed consent was obtained from all participants. The study was conducted in accordance with the Declaration of Helsinki.

The study included singleton pregnancies between the ages of 18 and 40. Gestational age was determined using the crown-rump length (CRL) measured during the first trimester ultrasound. A total of 240 cases were included in the study: 120 FGR cases (60 early-onset FGR, 60 late-onset FGR) and 120 healthy controls of appropriate gestational age.

The diagnosis of FGR was made according to the Delphi consensus criteria. It was defined as abdominal circumference (AC) or estimated fetal weight (EFW) below the 3rd percentile, or AC or EFW below the 10th percentile accompanied by Doppler findings. Early-onset FGR was defined as <32 weeks of gestation, and late-onset FGR as ≥32 weeks of gestation [18]. The control group consisted of pregnant women prospectively followed during the same study period who showed growth appropriate for their gestational age and had normal Doppler parameters. Controls were included consecutively to minimize selection bias. Selection bias was minimized through the prospective design, consecutive patient selection, and standardized measurement protocol.

The following conditions were excluded from the study: multiple pregnancy, maternal chronic systemic disease, fetal congenital or chromosomal anomaly, placental pathology, chronic medication use, smoking or alcohol use, failure to complete follow-up at the hospital or delivery at an external center, and incomplete clinical or ultrasonographic data.

All ultrasonographic examinations were performed by a single experienced operator (MAO) using a Voluson E10 ultrasound system (GE Healthcare, Zipf, Austria), equipped with a broadband 2–6 MHz convex transducer. The system provides high-resolution grayscale imaging as well as color and pulsed-wave Doppler capabilities, allowing detailed assessment of fetal cardiac morphology and venous hemodynamics. All measurements were obtained according to a standardized protocol to minimize measurement variability.

To assess intraobserver variability, a randomly selected subset of 30 examinations was re-evaluated by the same observer using stored ultrasound images at least two weeks after the initial measurements, blinded to the original results, in order to minimize recall bias. Intraobserver agreement was assessed using the intraclass correlation coefficient (ICC).

FO and RA transverse widths were measured in the apical four-chamber view, immediately above the atrioventricular valves, at end-systole, from inner edge to inner edge. Foramen ovale morphometry was evaluated using linear measurements (FO width) and proportional parameters (FO/RA ratio) obtained from the four-chamber view. The IVC diameter was measured from inner to inner at the point where the IVC was most clearly visible and the diameter was widest, at the level of the liver, in the long-axis view of the fetal abdomen in the sagittal plane. The measurement was performed based on the subcostal–sagittal long-axis approach, which is defined in the literature and most frequently used in fetal venous assessments. Z-scores for foramen ovale width and right atrial transverse diameter were calculated according to gestational age-specific reference ranges reported in the literature, whereas inferior vena cava diameter Z-scores were derived from published fetal venous nomograms [19,20]. IVC Doppler measurements were obtained from the segment proximal to the ductus venosus entrance and immediately before joining the right atrium. UA and IVC Doppler assessments were performed using the pulsed-wave Doppler method. The S/D ratio and pulsatility index (PI) for the UA, and the PI, preload index (PLI), and peak velocity index for veins (PVIV) for the IVC were recorded. The IVC preload index (PLI) was calculated as the ratio of the atrial contraction wave to the systolic forward flow; the PVIV was calculated using systolic, diastolic, and atrial wave velocities. All Doppler measurements were performed during periods of fetal immobility and absence of respiratory movements. The insonation angle was adjusted to be as parallel as possible to the flow direction and kept below 30°. At least three consecutive, homogeneous waveforms were recorded for each parameter, and mean values were used in the analyses.

The obstetric team delivering the birth and the neonatal team were not informed of the study measurements, and the study data were not shared in clinical decision-making processes. Neonatal and perinatal outcomes were recorded through the hospital information system in the postpartum period.

CAPO was defined as the presence of at least one of the following conditions: preterm birth, fetal distress, 5-min Apgar score < 7, or need for admission to the neonatal intensive care unit (NICU). Umbilical artery blood gas analysis was not routinely performed in all deliveries during the study period and was therefore not included in the analysis. To reduce subjectivity, Apgar scores were evaluated alongside objective perinatal outcomes, including fetal distress and neonatal intensive care unit admission, within the composite adverse perinatal outcome definition.

Sample size calculation was performed using G*Power software, version 3.1.9.7 (Heinrich Heine University, Düsseldorf, Germany). Based on previous fetal Doppler and cardiovascular studies, a moderate effect size was assumed (Cohen’s d = 0.5). With a two-sided alpha level of 0.05 and a targeted statistical power of 90%, the minimum required sample size was calculated as 86 participants per group for the primary comparison between the FGR and control groups. The final study population, consisting of 120 FGR cases and 120 gestational age-matched controls, exceeded this requirement, thereby ensuring adequate statistical power for the primary analyses.

### Statistical Analysis

The distribution of continuous variables was assessed using the Kolmogorov–Smirnov test, as the sample size in both groups was above 30. Continuous variables showing a normal distribution are presented as mean ± standard deviation, while those not showing a normal distribution are presented as median (interquartile range, IQR). Categorical variables were expressed as numbers and percentages (%). For comparisons between two independent groups (FGR and gestational age-matched control group), the independent samples *t*-test was used for continuous variables showing a normal distribution, and the Mann–Whitney U test was used for continuous variables not showing a normal distribution. The chi-square test or, where appropriate, Fisher’s exact test was used to compare categorical variables. Receiver operating characteristic (ROC) curve analysis was performed to evaluate the diagnostic performance of fetal cardiac morphometric parameters and IVC Doppler indices in predicting CAPO development. In ROC analyses, the area under the curve (AUC) was reported with a 95% confidence interval. The optimal cut-off values were determined using the Youden index (J = sensitivity + specificity − 1). Multivariate logistic regression analysis was applied to evaluate independent predictors for CAPO. Clinically meaningful variables consistent with the literature were included in the model: maternal age, body mass index (BMI), gestational age at ultrasound, FO/RA, and IVC PI. To allow for a more clinically meaningful interpretation of the odds ratios, the FO/RA ratio was multiplied by 10 before being included in the model, and the reported odds ratios were presented to represent a 0.1 unit increase in FO/RA. Results were reported as the odds ratio (OR) and 95% confidence interval (CI). Cases with missing clinical or ultrasonographic data were excluded from the analyses. All statistical analyses were performed using IBM SPSS Statistics for Windows, version 31.1 (IBM Corp., Armonk, NY, USA), and *p* < 0.05 was considered statistically significant. All statistical analyses were performed by the first author.

## 3. Results

A total of 240 pregnancies were included in the study, comprising 120 fetuses with FGR and 120 gestational age-matched appropriate-for-gestational-age controls. Maternal age, BMI, parity, and gestational age at ultrasonographic examination were comparable between the FGR and control groups (all *p* > 0.05). In contrast, gestational age at birth and birth weight were significantly lower in the FGR group (both *p* < 0.001). The incidence of fetal distress, NICU admission, and CAPO were higher among pregnancies complicated by FGR compared with controls, with statistically significant differences observed (all *p* < 0.001) (Table 1).

When FGR cases were stratified into EO-FGR and LO-FGR subgroups, each subgroup was compared separately with its gestational age-matched control group. In both EO-FGR and LO-FGR groups, gestational age at birth and birth weight were significantly lower than in their respective control groups (all *p* < 0.001). Moreover, the incidence of CAPO was significantly higher in both EO-FGR and LO-FGR fetuses compared with controls (both *p* < 0.001). In the EO-FGR subgroup, higher rates of preterm birth and NICU admission were observed compared with the corresponding control group (*p* = 0.010 and *p* < 0.001, respectively). In addition, fetal distress and lower Apgar scores at 1 and 5 min were more commonly observed in EO-FGR fetuses compared with controls, while fetal distress showed a non-significant trend toward higher frequency. Similarly, in the LO-FGR subgroup, fetal distress and reduced Apgar scores at both 1 and 5 min were also observed more frequently compared with the control group (all *p* < 0.05) (Table 2).

Intraobserver agreement for ultrasonographic measurements was good, with intraclass correlation coefficients exceeding 0.85 for foramen ovale and right atrium measurements and for inferior vena cava Doppler parameters.

Fetuses with FGR exhibited smaller FO width, lower FO Z-scores, reduced FO/RA ratios, and smaller IVC diameters compared with controls (all *p* < 0.05). IVC Z-scores were also significantly lower in the FGR group (*p* < 0.001). Umbilical artery Doppler parameters, including S/D ratio and PI, did not differ significantly between groups (Table 3). Venous Doppler indices demonstrated significant alterations in the FGR group. IVC PLI, IVC PI, and PVIV were all significantly higher in fetuses with FGR compared with controls (all *p* < 0.001) (Table 3).

Subgroup analysis revealed that both EO-FGR and LO-FGR fetuses had significantly smaller FO width and reduced IVC diameters compared with controls, while a lower FO/RA ratio was observed predominantly in the LO-FGR subgroup. Reductions in FO width and IVC diameter appeared to be more pronounced in the EO-FGR subgroup. IVC PI values were significantly elevated in both EO-FGR and LO-FGR groups, whereas IVC PLI and PVIV showed borderline or nonsignificant differences in the EO-FGR subgroup (Table 4).

**Figure 1:**
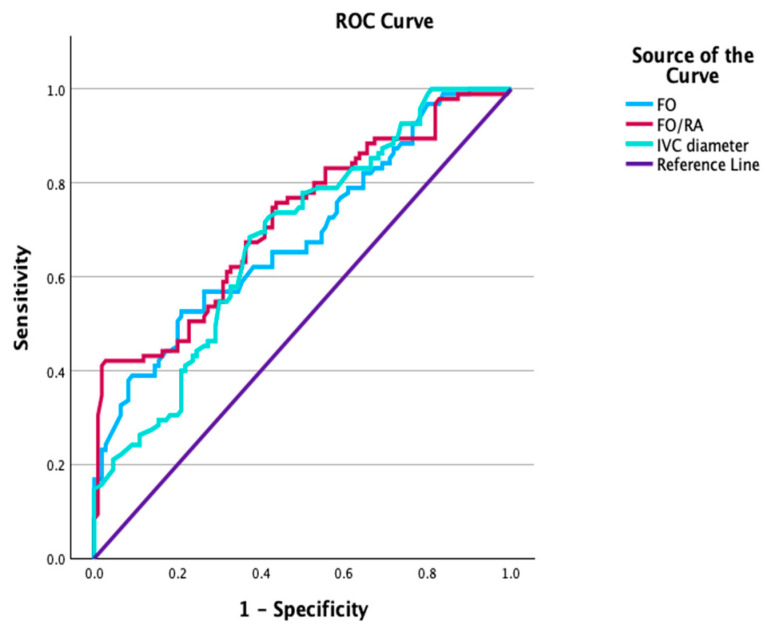
Receiver operating characteristic (ROC) curves for foramen ovale (FO) width, foramen ovale-to-right atrium (FO/RA) ratio, and inferior vena cava (IVC) diameter in predicting composite adverse perinatal outcome (CAPO) among fetuses with fetal growth restriction.

**Figure 2:**
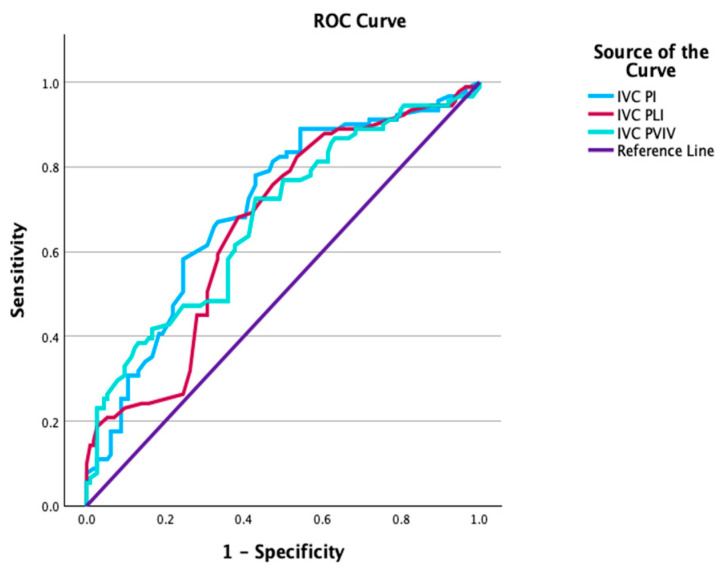
Receiver operating characteristic (ROC) curves of inferior vena cava (IVC) Doppler indices—pulsatility index (PI), preload index (PLI), and peak velocity index for veins (PVIV)—for predicting composite adverse perinatal outcome (CAPO) in fetuses with fetal growth restriction.

ROC curve analyses were performed within the FGR cohort to assess the ability of ultrasonographic parameters to predict CAPO. FO width demonstrated an area under the curve (AUC) of 0.701 (95% CI 0.614–0.759), with an optimal cut-off value of <5.21 mm. The FO/RA ratio showed the highest discriminative performance among the evaluated parameters (AUC 0.722, 95% CI 0.652–0.792), with a cut-off value of <0.41. Among venous parameters, IVC PI showed an AUC of 0.704 (95% CI 0.632–0.776) at a cut-off value of >1.29. IVC diameter, IVC PLI, and IVC PVIV also demonstrated moderate predictive performance for CAPO (Table 5, Figure 1 and Figure 2).

A multivariable logistic regression model including maternal age, BMI, gestational age at ultrasonographic examination, FO/RA ratio, and IVC PI was constructed to identify independent predictors of CAPO within the FGR cohort. After adjustment, FO/RA ratio and IVC PI remained independently associated with CAPO. A 0.1-unit increase in the FO/RA ratio was associated with a significantly reduced risk of CAPO (OR 0.57, 95% CI 0.39–0.82, *p* = 0.003), whereas higher IVC PI values were associated with an increased risk (OR 2.64, 95% CI 1.28–5.43, *p* = 0.008). Gestational age at ultrasonographic examination was also independently associated with CAPO (OR 0.90, 95% CI 0.81–0.99, *p* = 0.045). Maternal age and BMI were not independently associated with CAPO (Table 6).

## 4. Discussion

In our study, we evaluated the ultrasonographic characteristics of the left atrium (LA), right atrium (RA), and inferior vena cava (IVC) in fetuses diagnosed with fetal growth restriction (FGR) and assessed the relationship between these parameters and adverse perinatal outcomes. Our findings indicate that there are significant structural and hemodynamic changes in cardiac circulation in fetuses with FGR. The prominent findings in our study were that FO and IVC were proportionally smaller in the FGR group and that IVC Doppler patterns differed from those in controls. In particular, the significantly lower FO/RA ratio in fetuses with FGR and its clear association with perinatal outcomes suggest that FO morphometry may be an important indicator of fetal adaptation mechanisms. These findings may contribute to the noninvasive assessment of cardiovascular adaptation in FGR, in line with recent studies emphasizing the role of intracardiac morphometry in fetal cardiovascular remodeling [6,9,14].

Structural changes in the foramen ovale in fetuses with FGR are also well described in the literature. Kiserud and colleagues reported that, despite the relative preservation of right atrial diameter in growth-restricted fetuses, the diameter of the foramen ovale was significantly reduced [13]. It has been shown that this change can also be evident before 32 weeks of gestation and in the presence of severe placental insufficiency [13]. Although it has been suggested that the absolute reduction in the size of the foramen ovale may be a limiting factor in fetal circulation, it is known that this does not always mean that it disrupts the brain-sparing circulation. Indeed, in response to chronic hypoxia due to placental insufficiency, the most common cause of FGR, a brain-sparing mechanism develops, and cardiac output is prioritized for distribution to the brain and heart through the flow directed to the left heart via the foramen ovale [21]. The literature shows that in growth-restricted fetuses, although the foramen ovale is morphologically smaller, the high-oxygenated flow from the ductus venosus continues to be selectively directed to the left atrium through increased velocity and altered atrial pressure gradients, and that this is consistent with brain-sparing circulation [13,15,22]. The findings obtained in our study with a larger patient group support these pathophysiological mechanisms and suggest that the FO/RA ratio may be a meaningful indicator of adaptation in FGR.

The decrease in IVC diameter in the FGR group indicates a redistribution of fetal venous return. Within the framework of cerebral protective adaptation, blood flow in the hypoxic fetus is directed to the head and upper body; as a result, the superior vena cava dilates while the IVC diameter relatively decreases [23,24,25]. The IVC diameter narrowing in our findings is consistent with this phenomenon; under conditions of placental insufficiency, blood flow to the body and lower extremities decreases, while most of the oxygenated blood is directed to the upper body, resulting in the IVC vessel appearing smaller in diameter and having a reduced flow volume [26,27]. Consequently, IVC measurements emerge as a parameter reflecting the central redistribution of circulation in FGR.

The absence of significant deterioration in IVC Doppler parameters in the EO-FGR group may reflect that the examination was performed at a stage when fetal compensation was still preserved, rather than the absence of circulatory stress in these fetuses. It is known that venous Doppler changes are a late indicator of fetal cardiovascular decompensation rather than an early adaptive response and become more pronounced in advanced stages of hypoxia [28,29]. This concept is further supported by recent clinical and experimental studies highlighting venous Doppler alterations as markers of advanced fetal cardiovascular stress [6,9,17,25]. Therefore, in the EO-FGR group, it can be predicted that IVC Doppler findings may become more pronounced in examinations performed closer to delivery or in serial ultrasonographic evaluations. This finding suggests that early- and late-onset FGR cases are evaluated at different time windows of fetal circulation adaptation and emphasizes the importance of time-dependent evaluations in EO-FGR follow-up.

Another important finding in our study is that changes in the foramen ovale become apparent earlier compared to IVC Doppler findings. In particular, the fact that the FO/RA ratio was found to be significant in both the general FGR group and in subgroup analyses suggests that structural adaptations in the foramen ovale may be an early response to fetal hypoxia. Since the foramen ovale plays a key role in directing high-oxygenated blood from the ductus venosus to the left atrium [19], morphometric changes in this structure can be considered an early indicator of fetal circulation reorganization. In contrast, the literature reports that venous Doppler abnormalities reflect advanced stages of fetal stress where compensatory mechanisms are exhausted [28,29]. Therefore, our study suggests that FO morphometric changes may reflect an early adaptive response, while IVC Doppler findings may reflect later hemodynamic deterioration.

Consideration of early-onset and late-onset FGR groups provides important clues regarding the timing of hemodynamic adaptation. Ultrasound evaluation in our EO-FGR group was performed at an average of 31–32 weeks of gestation, while the LO-FGR group was examined at approximately 36 weeks (in accordance with the diagnosis of placental insufficiency and the follow-up process). This time difference may have affected the severity of the findings. Early-onset FGR cases may theoretically develop more pronounced cardiac changes because they are exposed to hypoxia for a longer period; indeed, Kiserud et al. reported that reduction in FO can be seen as early as the beginning of the 30 s in the presence of severe placental insufficiency [13]. However, the gestational age of our EO-FGR group at the time of examination may have coincided with a period when venous circulation changes had not yet fully established. Since venous Doppler changes tend to appear in the later stages of fetal hypoxia [28,29], it is conceivable that IVC Doppler findings in the EO-FGR group would be more pronounced if the fetuses were evaluated closer to term or in more advanced stages of hypoxia. Recent longitudinal studies have similarly emphasized the time-dependent nature of fetal cardiovascular adaptation in early- and late-onset FGR [6,17]. In contrast, since LO-FGR cases were evaluated in the near-term weeks, the hemodynamic effects of placental insufficiency (e.g., cerebral sparing and increased venous pulsatility) may have been captured at the time of measurement when they were already pronounced. This suggests that the early and late forms of FGR are evaluated in different time windows of similar pathophysiological processes. In early-onset cases, compensatory mechanisms may still be functioning at full capacity during the examination, whereas in late-onset cases, the fetus may be closer to its adaptive limits.

The most important strengths of our study are its prospective design, the separate evaluation of early- and late-onset cases in a well-defined FGR cohort, and the combined consideration of foramen ovale morphometry and venous Doppler parameters. The use of proportional measurements, such as the FO/RA ratio, has increased the clinical interpretability of the findings by reducing the effect of fetal size and gestational age differences. Furthermore, the evaluation of perinatal outcomes using multivariate logistic regression analysis supports the independence of the identified associations.

However, our study has some limitations. Venous Doppler assessments were performed at a single time point, and longitudinal follow-up data are not available. The limited venous Doppler findings, particularly in the early-onset FGR group, may be related to the timing of the examination, which may have preceded the development of overt fetal cardiovascular decompensation. The absence of serial venous Doppler assessments limits direct evaluation of the temporal evolution of fetal cardiovascular compensation, and our interpretations should therefore be considered hypothesis-generating. Evaluations performed closer to delivery or with serial measurements may better elucidate the temporal progression of venous hemodynamic changes. In addition, all ultrasonographic examinations were performed by a single experienced operator to ensure methodological consistency and minimize interobserver variability, given the operator-dependent nature of fetal cardiac morphometry and venous Doppler measurements. While this approach strengthens internal validity, it may limit the external generalizability of the findings to multicenter or routine clinical settings. Finally, although preterm birth may be medically indicated as part of clinical management, it was deliberately included in the composite adverse perinatal outcome definition as a clinically meaningful consequence of impaired fetal well-being rather than as a confounding effect of management decisions.

Therefore, future multicenter studies involving multiple operators and standardized acquisition protocols are warranted to confirm the reproducibility and broader applicability of these results.

## 5. Conclusions

This study demonstrates that foramen ovale morphometry and inferior vena cava hemodynamics reflect complementary stages of fetal cardiovascular adaptation in fetal growth restriction. A reduced foramen ovale-to-right atrium ratio may represent an early adaptive response to chronic fetal hypoxia, whereas alterations in inferior vena cava Doppler parameters appear to reflect more advanced hemodynamic compromise. The observed differences between early- and late-onset FGR highlight the importance of considering the timing of fetal assessment when interpreting venous and intracardiac parameters. Together, these findings suggest that combined evaluation of FO morphometry and IVC Doppler may provide additional value in perinatal risk stratification and support further longitudinal and multicenter studies to clarify their role in clinical surveillance.

## Figures and Tables

**Table 1:** Maternal and perinatal characteristics in FGR and control groups.

	Total FGR n = 120	Control n = 120	*p* Value
Maternal age (years)	27.0 (7.0)	28.0 (7.0)	0.776
Nulliparous	48 (40.0%)	52 (43.3%)	0.356
BMI (kg/m^2^)	28.13 (6.03)	29.75 (5.64)	0.276
Early-onset FGR n (%)	60 (50.0%)		
Late-onset FGR n (%)	60 (50.0%)		
Gestational age at USG examination (weeks)	34.0 (5.0)	33.3 (3.5)	0.154
Gestational age at birth	37.0 (1)	38.2 (2)	**<0.001**
Mode of Delivery			0.641
Vaginal delivery	44 (36.6%)	42 (35.0%)	
Primary cesarean section	35 (29.2%)	30 (25.0%)	
Repeat cesarean section	41 (34.2%)	48 (40.0%)	
Birth weight (grams)	2280 (403)	3150 (468)	**<0.001**
Estimated Fetal Weight percentile	4.15 (7.6)	36.5 (23.9)	**<0.001**
Preterm birth	28 (23.3%)	14 (11.6%)	0.136
Fetal Distress	28 (23.3%)	9 (7.5%)	**<0.001**
Apgar score at 1st minute	8 (2)	9 (1)	**<0.001**
Apgar score at 5th minute	9 (2)	9 (1)	**<0.001**
NICU admission	40 (33.3%)	15 (12.5%)	**<0.001**
CAPO	61 (50.8%)	27 (22.5%)	**<0.001**

Note: Data are expressed as n (%) or median (IQR) where appropriate. A *p* value of <0.05 indicates a significant difference, and statistically significant *p* values are in bold. Early- and late-onset FGR subgroups are presented descriptively in Table 1, while detailed comparisons are shown in Table 2. Abbreviations: BMI, body mass index; FGR, fetal growth restriction; NICU, neonatal intensive care unit; CAPO, composite adverse perinatal outcome.

**Table 2:** Maternal and perinatal characteristics in early- and late-onset fetal growth restriction subgroups and controls.

	LO-FGR n = 60	Control n = 60	*p* Value	EO-FGR n = 60	Control n = 60	*p* Value
Maternal age (years)	27.17 ± 5.36	28.01 ± 4.79	0.870	27.68 ± 4.85	27.55 ± 4.26	0.304
Nulliparous	28 (46.6%)	28 (46.6%)	1.000	20 (33.3%)	24 (40.0%)	0.450
BMI (kg/m^2^)	28.17 ± 4.44	29.88 ± 3.56	0.306	29.60 ± 4.55	28.82 ± 3.74	0.755
Gestational age at diagnosis (weeks)	35.6 (2.5)			29.2 (4.4)		
Gestational age at USG examination (weeks)	36.0 (1.5)	35.5 (2.0)	0.120	31.1 (3.2)	31.6 (1.6)	0.156
Gestational age at birth	37.1 (0.4)	38.2 (1.2)	**<0.001**	37 (1.6)	38.5 (3.0)	**<0.001**
Mode of Delivery			0.178			**0.017**
Vaginal delivery	26 (43.3%)	23 (38.3%)		18 (30.0%)	19 (31.7%)	
Primary cesarean section	15 (25.0%)	17 (28.4%)		20 (33.3%)	13 (21.6%)	
Repeat cesarean section	19 (31.7%)	20 (33.3%)		22 (36.7%)	28 (46.7%)	
Birth weight (grams)	2350 (350)	3180 (510)	**<0.001**	2100 (770)	3240 (460)	**<0.001**
Preterm birth	8 (13.3%)	6 (10.0%)	0.381	20 (33.3%)	8 (13.3%)	**0.010**
Fetal Distress	13 (21.6%)	2 (3.3%)	**<0.001**	15 (25.0%)	7 (11.6%)	0.062
Apgar score at 1st minute	8 (2)	9 (1)	**0.009**	7 (2)	9 (1)	**<0.001**
Apgar score at 5th minute	9 (2)	9 (1)	**0.029**	8 (3)	10 (1)	**<0.001**
NICU admission	13 (21.6%)	7 (11.6%)	0.063	27 (45.0%)	8 (13.3%)	**<0.001**
CAPO	28 (46.6%)	13 (21.6%)	**<0.001**	33 (55.0%)	14 (23.3%)	**<0.001**

Note: Data are expressed as n (%), mean ± SD, or median (IQR) where appropriate. A *p* value of <0.05 indicates a significant difference, and statistically significant *p* values are in bold. *p* values indicate comparisons with gestational age-matched controls; non-applicable variables were not tested (Gestational age at diagnosis). Abbreviations: EO-FGR, early onset fetal growth restriction; LO-FGR, late-onset fetal growth restriction; BMI, body mass index; USG, ultrasonography; NICU, neonatal intensive care unit; CAPO, composite adverse perinatal outcome.

**Table 3:** USG findings in FGR compared to controls.

	Total FGR n = 120	Control n = 120	*p* Value
FO width (mm)	5.10 (1.35)	5.32 (1.23)	**<0.001**
FO Z score	−0.73 (1.23)	0.55 (0.86)	**0.042**
RA width (mm)	12.20 (2.5)	13.95 (2.65)	0.061
RA Z-score	−2.45 (2.25)	−0.55 (0.89)	**0.016**
FO/RA	0.40 (0.10)	0.42 (0.15)	**0.021**
IVC diameter (mm)	3.62 (0.96)	4.31 (0.99)	**<0.001**
IVC Z-score	−0.64 (1.27)	0.56 (1.36)	**<0.001**
UA S/D	2.62 (0.88)	2.58 (0.49)	0.471
UA PI	0.99 (0.26)	0.94 (0.19)	0.181
UA PI percentile	60.0 (62.0)	47.0 (51.0)	0.424
IVC PLI	0.78 (0.13)	0.72 (0.15)	**<0.001**
IVC PI	1.38 (0.40)	1.14 (0.48)	**<0.001**

Note: Data are expressed as n (%), mean ± SD, or median (IQR) where appropriate. A *p* value of < 0.05 indicates a significant difference, and statistically significant *p* values are in bold. Non-normally distributed continuous variables were compared using the Mann–Whitney U test. Abbreviations: FGR, fetal growth restriction; FO: foramen ovale, RA: right atrium; FO/RA: foramen ovale-to-right atrium ratio; IVC: inferior vena cava; UA: umbilical artery; PI: pulsatility index; PLI: Preload index; PVIV: pulsatility index for veins.

**Table 4:** USG findings in early- and late-onset fetal growth restriction subgroups and controls.

	LO-FGR n = 60	Control n = 60	*p* Value	EO-FGR n = 60	Control n = 60	*p* Value
FO width (mm)	5.45 (0.98)	5.78 (1.13)	**0.008**	4.79 (1.62)	5.14 (0.86)	**0.002**
FO Z score	−0.38 (0.88)	−0.25 (0.85)	0.543	−0.94 (1.48)	−0.59 (0.73)	**0.005**
RA width (mm)	13.14 (2.17)	14.15 (2.67)	0.165	11.33 (2.11)	13.80 (2.64)	**0.002**
RA Z-score	−1.58 (0.96)	−0.54 (1.04)	0.123	−3.58 (2.46)	−0.57 (1.03)	**<0.001**
FO/RA	0.41 (0.08)	0.45 (0.14)	**0.023**	0.39 (0.13)	0.40 (0.15)	0.188
IVC diameter (mm)	3.77 (0.98)	4.39 (1.06)	**0.004**	3.50 (1.09)	4.27 (1.12)	**<0.001**
IVC Z-score	−0.70 (1.14)	0.30 (1.03)	**<0.001**	−0.38 (1.08)	0.68 (1.50)	**<0.001**
UA S/D	2.46 (0.65)	2.48 (0.48)	0.412	2.89 (1.20)	2.73 (0.74)	0.471
UA PI	0.91 (0.25)	0.89 (0.16)	0.236	1.04 (0.38)	0.99 (0.20)	0.181
UA PI percentile	56.0 (61.0)	47.0 (53.0)	**0.062**	63.0 (74.0)	48.5 (51.0)	0.474
IVC PLI	0.81 (0.12)	0.70 (0.15)	**<0.001**	0.75 (0.15)	0.73 (0.14)	0.067
IVC PI	1.41 (0.61)	1.17 (0.54)	**<0.001**	1.33 (0.47)	1.12 (0.41)	**0.005**
IVC PVIV	1.69 (0.96)	1.29 (0.90)	**<0.001**	1.45 (1.04)	1.40 (0.13)	0.059

Note: Data are expressed as n (%), mean ± SD, or median (IQR) where appropriate. A *p* value of <0.05 indicates a significant difference, and statistically significant *p* values are in bold. Abbreviations: EO-FGR, early-onset fetal growth restriction; LO-FGR, late-onset fetal growth restriction; FO: foramen ovale, RA: right atrium; FO/RA: foramen ovale-to-right atrium ratio; IVC: inferior vena cava; UA: umbilical artery; PI: pulsatility index; PLI: Preload index; PVIV: pulsatility index for veins.

**Table 5:** ROC-based prognostic performance of ultrasound parameters for predicting composite adverse perinatal outcome within the FGR cohort.

	Cut-Off	Sensitivity (%)	Specificity (%)	AUC	95% CI	*p*-Value
FO width	<5.21	62.1%	62.2%	0.701	0.614–0.759	**<0.001**
FO/RA	<0.41	67.4%	64.6%	0.722	0.652–0.792	**<0.001**
IVC diameter	<3.89	68.4%	63.7%	0.682	0.609–0.759	**<0.001**
IVC PI	>1.29	67.0%	66.7%	0.704	0.632–0.776	**<0.001**
IVC PLI	>0.75	68.1%	62.4%	0.662	0.588–0.737	**<0.001**
IVC PVIV	>1.45	61.5%	63.3%	0.676	0.602–0.750	**<0.001**

Note: Bold values indicate statistically significant results (*p* < 0.05). FO: foramen ovale; FO/RA: foramen ovale-to-right atrium ratio; IVC: inferior vena cava; PI: pulsatility index; PLI: Preload index; PVIV: pulsatility index for veins. Cut-off values were found according to Youden index.

**Table 6:** Multivariate logistic regression model for predicting CAPO.

Variables	OR (95% CI)	*p* Value
Maternal age (years)	1.02 (0.96–1.08)	0.501
BMI (kg/m^2^)	1.02 (0.94–1.09)	0.603
GA at USG examination (weeks)	0.90 (0.81–0.99)	**0.045**
FO/RA ratio	0.57 (0.39–0.82)	**0.003**
IVC PI	2.64 (1.28–5.43)	**0.008**

FO/RA was multiplied by 10 prior to regression analysis; reported odds ratios represent a 0.1-unit increase in the FO/RA ratio. BMI: body mass index; GA: gestational age; FO/RA: foramen ovale-to-right atrium ratio; IVC: inferior vena cava; PI: pulsatility index.

## Data Availability

The datasets generated and analyzed during the current study are available from the corresponding author upon reasonable request, subject to institutional and ethical restrictions.

## Abbreviations

The following abbreviations are used in this manuscript:

AUC	Area Under the Curve
BMI	Body Mass Index
CAPO	Composite Adverse Perinatal Outcome
CI	Confidence Interval
EFW	Estimated Fetal Weight
EO-FGR	Early-Onset Fetal Growth Restriction
FO	Foramen Ovale
FO/RA	Foramen Ovale-to-Right Atrium Ratio
FGR	Fetal Growth Restriction
GA	Gestational Age
IVC	Inferior Vena Cava
LO-FGR	Late-Onset Fetal Growth Restriction
NICU	Neonatal Intensive Care Unit
OR	Odds Ratio
PI	Pulsatility Index
PLI	Preload Index
PVIV	Peak Velocity Index for Veins
RA	Right Atrium
ROC	Receiver Operating Characteristic
UA	Umbilical Artery
